# Imaging white matter microstructure with gradient‐echo phase imaging: Is ex vivo imaging with formalin‐fixed tissue a good approximation of the in vivo brain?

**DOI:** 10.1002/mrm.29213

**Published:** 2022-03-28

**Authors:** Kwok‐Shing Chan, Renaud Hédouin, Jeroen Mollink, Jenni Schulz, Anne‐Marie van Cappellen van Walsum, José P. Marques

**Affiliations:** ^1^ Donders Institute for Brain, Cognition and Behavior Radboud University Nijmegen The Netherlands; ^2^ Empenn, INRIA, INSERM, CNRS Université de Rennes 1 Rennes France; ^3^ Department of Medical Imaging, Anatomy, Donders Institute for Brain, Cognition and Behavior Radboud University Medical Center Nijmegen The Netherlands

**Keywords:** ex vivo imaging, microstructure, phase imaging, quantitative susceptibility imaging, white matter

## Abstract

**Purpose:**

Ex vivo imaging is a commonly used approach to investigate the biophysical mechanism of orientation‐dependent signal phase evolution in white matter. Yet, how phase measurements are influenced by the structural alteration in the tissue after formalin fixation is not fully understood. Here, we study the effects on magnetic susceptibility, microstructural compartmentalization, and chemical exchange measurement with a postmortem formalin‐fixed whole‐brain human tissue.

**Methods:**

A formalin‐fixed, postmortem human brain specimen was scanned with multiple orientations to the main magnetic field direction for robust bulk magnetic susceptibility measurement with conventional quantitative susceptibility imaging models. White matter samples were subsequently excised from the whole‐brain specimen and scanned in multiple rotations on an MRI scanner to measure the anisotropic magnetic susceptibility and microstructure‐related contributions in the signal phase and to validate the findings of the whole‐brain data.

**Results:**

The bulk isotropic magnetic susceptibility of ex vivo whole‐brain imaging is comparable to in vivo imaging, with noticeable enhanced nonsusceptibility contributions. The excised specimen experiment reveals that anisotropic magnetic susceptibility and compartmentalization phase effect were considerably reduced in the formalin‐fixed white matter specimens.

**Conclusions:**

Formalin‐fixed postmortem white matter exhibits comparable isotropic magnetic susceptibility to previous in vivo imaging findings. However, the measured phase and magnitude data of the fixed white matter tissue shows a significantly weaker orientation dependency and compartmentalization effect. Alternatives to formalin fixation are needed to better reproduce the in vivo microstructural effects in postmortem samples.

**Abbreviations**
B0: Static magnetic field
CC: Corpus callosum
COSMOS: Calculation of susceptibility through multiple orientation sampling
CST: Corticospinal tract
GM: Gray matter
GRE: Gradient echo
LBV: Laplacian boundary value (LBV) method
QUASAR: Quantitative susceptibility and residual mapping
SEGUE: A Speedy rEgion‐Growing Algorithm for Unwrapping Estimated Phase
SEPIA: Susceptibility mapping pipeline tool for phase images
Tacq: Acquisition time
V1: Principal diffusion direction
WM: White matter

## 1 INTRODUCTION

Quantitative susceptibility mapping is a physics‐driven method for studying the magnetic properties of biological tissues.^1^ One major challenge facing QSM research is to understand the gradient‐echo (GRE) signal phase evolution mechanism in white matter (WM).^2^, ^3^ In deep gray matter (GM), strong correlations between the isotropic magnetic susceptibility χ_i_ and iron concentration have been demonstrated.^4^ In WM, however, the abundance of diamagnetic myelin (relative to water) would indicate a strong QSM contrast relative to CSF.^2^, ^3^ The lack of this contrast has been attributed to various biophysical phenomena.^5^, ^6^ The lipid‐rich myelin bilayer sheath encapsulating the highly ordered axons leads to anisotropic magnetic susceptibility, χ_a_.^5^, ^6^, ^7^, ^8^, ^9^, ^10^ Water protons reside in different microstructural environments,^11^ namely myelin, intra‐axonal and extra‐axonal space, with different concentrations, relaxation properties and frequency shifts, resulting in microstructure orientation–dependent signal evolution.^12^, ^13^, ^14^, ^15^ The chemical exchange of protons between macromolecules and water can also introduce a frequency shift.^16^, ^17^ Understanding the WM phase‐contrast mechanisms can help us account for their impact on QSM.

Data with multiple orientations with respect to the main magnetic field direction (B_0_) are needed to investigate the orientation‐dependent WM phase contrast. Subject compliance limits the angular range achievable in vivo. Ex vivo imaging does not suffer from this limitation and allows both longer scanning sessions and histology to validate any microstructural findings.^18^, ^19^ A potential disadvantage of ex vivo experiments is the structural alteration associated with sample preparation, including tissue autolysis and formalin fixation. Significant differences in MR measurement parameters between ex vivo and in vivo imaging have been reported in DWI^20^ and single/multi‐compartment relaxometry.^21^, ^22^, ^23^, ^24^ Conversely, χ_i_ of brain tissue does not change significantly between in vivo and ex vivo conditions, neither in iron‐rich nor myelin‐rich regions,^25^, ^26^ although the origins of the susceptibility contrast can be different.^26^

In this study, we evaluate the effects of magnetic susceptibility, compartmentalization, and chemical shift on the MR phase signal in WM with a formalin‐fixed, postmortem human brain sample at 3 T, providing insights into the use of fixed tissue in future QSM research. Multiple orientation experiments were performed on both whole‐brain and excised tissue samples, enabling both traditional QSM maps, ground‐truth susceptibility measurements, and a separate calculation of the microstructure compartmentalization information.

## 2 METHODS

### 2.1 Tissue processing

A postmortem human brain of a donor with no neurological disorder history (male; 78 years; cause of death: myocardial infarction) was used for this work with permission from the local ethics committee. After extracting the brain from the skull, it was immersed in 5% formalin fixation solution and stored at room temperature (postmortem interval: 10 h). Detailed description of the fixation procedure and the fixative chemical composition can be found in Doomernik et al.^27^

**FIGURE 1:**
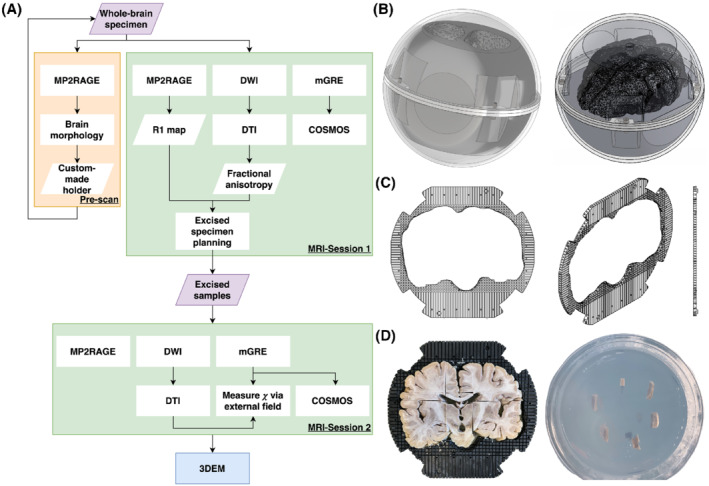
(A) Summary of this study consisting of a prescan, two MRI sessions, and a 3D electron microscopy (3DEM) session. (B) Schematic diagrams of the experimental setup used in the first MRI session. The setup is made of two parts: an outer transparent sphere that allows the specimen to rotate freely, and a tailor‐made inner holder to ensure the specimen was in a fixed position within the sphere. (C) Schematic diagrams of the plate that forms the inner holder. The center of the plate is a space with the shape of the specimen, surrounded by a grid structure providing a location reference in the MR image (the gaps were filled with water, whereas the plate material gave no detectable signal), and a guide during the sample excision for the second imaging session. (D) An illustration of how the samples were acquired with the aid of the plate (right) and were embedded in the agar inside the cylindrical container (left). Abbreviations: COSMOS, calculation of susceptibility through multiple orientation sampling; GRE, gradient echo

The study workflow is summarized in Figure 1A. After 1 month of fixation, the specimen was prescanned to create a tailor‐made holder that prevented dislocation of the specimen during imaging. The holder consists of a stack of 35 4‐mm‐thick, 3D‐printed plastic plates with a cavity in the middle having the same shape as the brain (see Figure 1B,C), where the specimen can be fitted tightly into the holder and the holder was held in place with a surrounding spherical container. Each plate had a grid layout (element: 4 × 4 mm) that provided landmarks in MRI images for planning and guiding the tissue excision for the validation experiment in the second MRI session. Further details on the holder and its use can be found in Supporting Information Section 1.

The first MRI session was performed after the fixation time of 5 months. Before the start of the first session, the sample was kept overnight under demineralized water at low pressure (10 mbar), followed by a continuous rotation of the sample for 30 min to remove residual air bubbles in the ventricles before inserting it into the holder. Demineralized water was used as the liquid medium in the first session.

After the first MRI session, the whole‐brain specimen was stored in a refrigerator at 2°C before the tissue excision was performed. Ten WM specimens from the corpus callosum and corticospinal tracts (fiber population dominated by one main direction) with excisable volume (≥ 2 elements of the holder plate grid [ie, 128 mm^3^]) were obtained from the whole‐brain specimen. These WM samples and two additional deep GM samples (globus pallidus and putamen) were embedded in 1% low‐gelling temperature agarose (A9414; Sigma Aldrich, Germany, with demineralized water) to avoid tissue denaturation between the scans. The samples were placed in a cylindrical polymethyl methacrylate container, with their long axes perpendicular to the cylindrical axis (see Figure 1D and Supporting Information Section 1.2). Imaging was performed 1 day after excising the samples and 5 days after the whole‐brain scan. Finally, 3D electron microscopy was used to examine two CC samples, providing histological references to MRI data (see Supporting Information Section 4).

### 2.2 Magnetic resonance imaging experiments

#### 2.2.1 Data acquisition

The study was approved by the local ethics committee. All MRI data were acquired on a 3T scanner (Prisma, Siemens, Germany) at room temperature (20°C) using a 64‐channel array coil.

An MP2RAGE sequence adapted to short T_1_ values (250–1000 ms) was used for the prescan session to obtain the specimen morphology with the parameters: TI_1_/TI_2_/TR = 311/1600/3000 ms, α_1_/α_2_ = 4°/6°, 1‐mm isotropic resolution, and Tacq = 5 min.

For the whole‐brain scanning session, the following protocol was used:

1. Same MP2RAGE as in pre‐scan;
2. Monopolar, 3D multi‐echo GRE, 1‐mm isotropic resolution, TR/TE_1_/ΔTE/TE_6_ = 40/3.45/6.27/34.8 ms, α = 20° (optimized for WM T_1_), 10 orientations to the B_0_ direction, chosen to optimize microstructural information decoding^28^; Tacq = 10.3 min/orientation; and
3. 2D spin‐echo EPI DWI, 1.6‐mm isotropic resolution, TR/TE = 15 241/77.6 ms, two shells (b = 0/1250/2500 s/mm^2^, 17/120/120 diffusion‐encoding directions with seven b = 0 measurements collected with reversed phase‐encode blips for distortion correction), Tacq = 17 min/repetition (only performed at the last GRE orientation with 20 repetitions).

In the second scanning session, the excised specimens were scanned using the following protocol:

1. Same MP2RAGE as in prescan;
2. Monopolar, 3D multi‐echo GRE, 0.7‐mm isotropic resolution, TR/TE_1_/ΔTE/TE_9_ = 39.2/2.05/4.23/35.89 ms, α = 15° (optimized for agar T_1_), 10 orientations to the B_0_ direction (randomized acquisition order, see Supporting Information S2), Tacq = 31.5 min/orientation; and
3. 2D spin‐echo EPI DWI, 1‐mm isotropic resolution, TR/TE = 15 241/77.6 ms, same diffusion encoding scheme as in the first session, and Tacq = 1.2 h/repetition (only performed after GRE with nine repetitions).

Detailed information on the rotation angles used can be found in the Supporting Figures S2 and S3.

#### 2.2.2 Data processing

Each DWI repetition was preprocessed separately with the Marchenko‐Pastur principal component analysis denoising,^29^ susceptibility‐induced distortion correction,^30^, ^31^ and eddy current–induced distortion correction.^32^ After averaging, DTI was performed to extract the principal diffusion direction (V1) using FSL's FDT.^33^ The brain mask and the excised specimen mask were obtained on the R_1_ map and the DWI data using semi‐automatic segmentation on ITK‐snap.^34^ The R_1_ map, DTI results, and associated signal masks were then registered to the GRE data using rigid body transform with linear interpolation.^35^ For the analyses of the excised specimens, we assumed axons within each sample had a single fiber orientation given by the mean V1 across the sample mask.

All GRE data were first corrected for the gradient nonlinearity–induced distortions. Image registration was performed to align the GRE data from all orientations to a common space (first session: a space for standard visualization and independent of the experiment rotations; second session: the GRE space at position #6) using rigid body transform and linear interpolation. The B_0_ direction of each orientation was subsequently rotated using the transformation matrix resulting from image registration. The R_2_* maps were computed with a closed‐form solution.^36^ Field maps were computed in SEPIA^37^ with SEGUE^38^ and optimum weighted‐echo combination,^39^ and background fields were removed using LBV.^40^ The value of χ_i_ was derived using calculation of susceptibility through multiple orientation sampling (COSMOS).^41^ Additionally, quantitative susceptibility and residual (QUASAR) was applied to test whether $\chi_{i}$ improved when nonsusceptibility contributions (f_ρ_) to the field were simultaneously estimated^42^:

(1) $$f_{N}=d_{N}*\chi +f_{\rho }$$

where f_N_ and d_N_ are the tissue field and a unit dipole field associated with orientation N, and * is the convolution operator.

For the excised specimen data, in addition to COSMOS, the quantification of χ_i_ and χ_a_ of the sample without confounding with nonsusceptibility microstructural contributions was performed by fitting the external field f_N_ on the agar surrounding the specimen in the following way^43^:

(2) $$\min_{\chi_{i}\text{\&}\chi_{a}}\left\|M_{\text{agar}}\left(f_{N}-\chi_{i}\delta f_{i,N}-\chi_{a}\delta f_{a,N}-C_{N}\right)\right\|$$

where M_agar_ is the binary mask on agar with inner and outer boundaries 1 and 5 voxels away from the specimen tissue boundary in all directions; δf_i,N_ and δf_a,N_ are the frequency perturbations generated by a specimen per units of χ_i_ and χ_a_, which are determined by the B_0_ direction at orientation N and the angle *θ* between the DTI‐derived specimen fiber orientation and the B_0_ direction; and C_N_ accounts for any baseline frequency differences in agar due to chemical exchange in the agar or residual background fields for a particular orientation. Linear regression was used to compare the susceptibility measurements between COSMOS and the external field method and between the two imaging sessions.

Tissue compartmentalization contributions to the MR phase can be measured as the residual field (f_R_) inside the specimen with mask M_specimen_ from the external field measurement:

(3) $$f_{R,N}=\bar{M_{\text{specimen}}\left(f_{N}-\chi_{i}\delta f_{i,N}-\chi_{a}\delta f_{a,N}\right)}$$

which is expected to vary with *θ* between the specimen fiber direction and B_0_ as

(4) $$f_{R,N}=A\sin^{2}\theta_{N}+B$$

where A explains the microstructure orientation‐dependent effect and B is orientation‐invariant, associated with both magnetization exchange and microstructure.^43^

## 3 RESULTS

**FIGURE 2:**
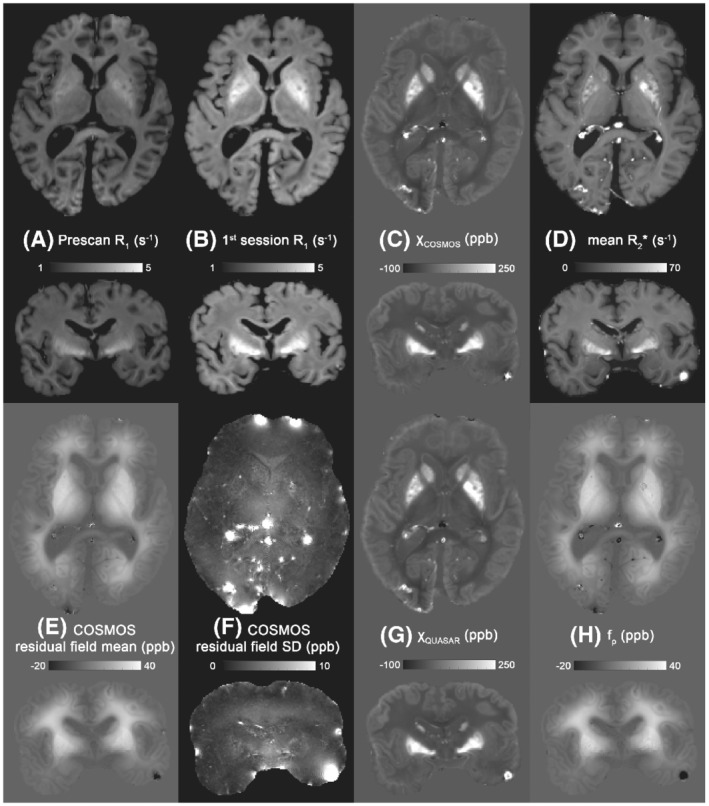
Quantitative maps of the whole‐brain specimens in transverse and coronal directions. (A) R_1_ map obtained from the prescan after formalin fixation of 1 month. (B) R_1_ map obtained from the first imaging session while the brain was fixed for 5 months. (C) COSMOS‐derived χ_i_. (D) Mean R_2_* map across the 10 rotations. (E,F) Mean and SD of the residual fields from COSMOS across orientations. (G,H) Quantitative susceptibility and residual (QUASAR)–derived χ_i_ and nonsusceptibility contribution map

Whole‐brain results are shown in Figure 2. The R_1_ maps obtained from the first session show faster relaxation rates than those from the prescan, with the contrast between WM and cortical GM being clearly reduced, and the basal ganglia showing an increased R_1_ (Figure 2A,B). The χ_i_ of COSMOS is consistent with previously published in vivo data (see Figure S4), where an opposite contrast between WM and GM can be observed (Figure 2C). However, the residual field of COSMOS shows a slowly varying pattern throughout the brain (Figure 2E) and is relatively stable across all orientations (Figure 2F). This residual map shares similar contrasts and values with the QUASAR nonsusceptibility contributions map (Figure 2H), and the χ_i_ derived from QUASAR (Figure 2G) and COSMOS (Figure 2C) are comparable. Susceptibility tensor imaging was also performed, but the results beyond the mean susceptibility were not informative.

**FIGURE 3:**
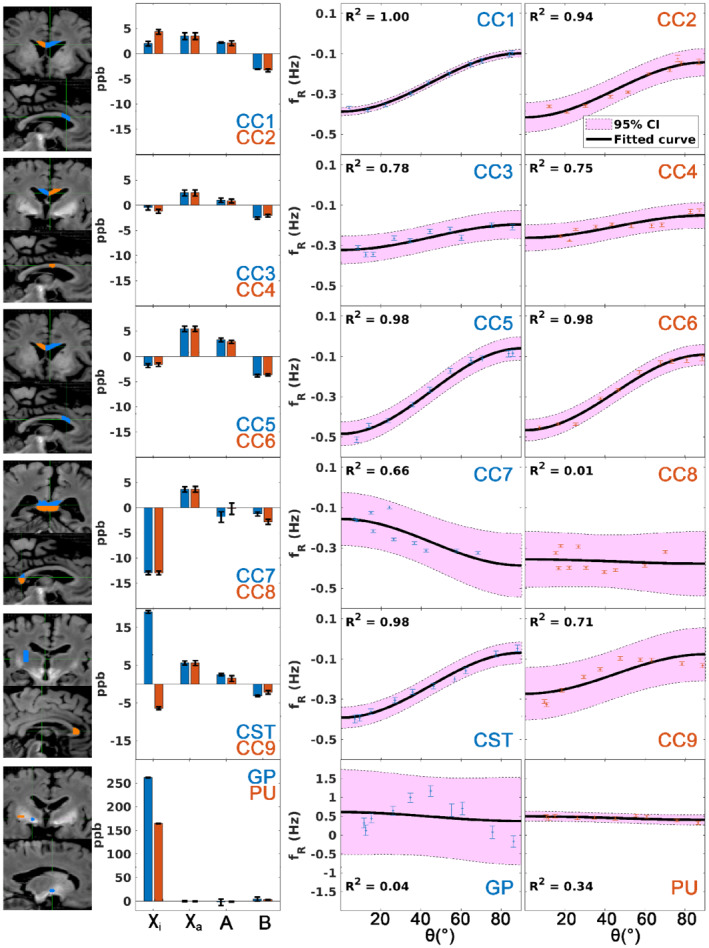
(From left to right) The corresponding regions of interest of the excised specimens in the whole‐brain R_1_ map (colors match the results on the right); bar plots of the isotropic and anisotropic magnetic susceptibility and coefficients A and B of the fitting of sin^2^ *θ*; and sin^2^ *θ* fittings that reflect microstructure compartmentalization of the excised specimens. Each row shows the results of two specimens. Error bar indicates the SEM (except for the coefficients A and B in the bar plots, which represents the 95% confidence interval [CI] in this case). The shaded region in the sin^2^ *θ* fittings corresponds to the 95% CI. Abbreviations: CC, corpus callosum; CST, corticospinal tract; GP, globus pallidus; PU, putamen

Figure 3 shows the microstructure measurements of the excised specimens using the external field on agar, with their corresponding regions of interest overlaid on the whole‐brain R_1_ map. The mean χ_i_ and χ_a_ are 1.17 ± 9.18 ppb and 4.03 ± 1.63 ppb across all WM specimens. A relatively strong positive χ_i_ is found in the corticospinal tract specimen (19.17 ppb), which was found retrospectively coming from a remnant of globus pallidus at one end of the excised sample. The coefficient A of sin^2^ *θ* dependence reflecting the WM microstructure effect has a mean value of 1.46 ± 1.55 ppb with a mean intercept B of −2.75 ± 0.79 ppb in WM. However, the R^2^ of the residual field fitting suggests that not all WM specimens fit the sin^2^ *θ* function equally well, especially the genu and the splenium of the corpus callosum (CC) have the lowest R^2^ (ranging from 0.01 to 0.71) among the WM samples. This is attributed to both the higher fiber dispersion^44^ and the bending of the fibers as they cross the middle of the bundle, invalidating the single fiber direction assumption. Therefore, we focused on the six WM specimens obtained from the body of the CC (CC1‐CC6) compared with the whole‐brain data in the linear regression analysis.

**FIGURE 4:**
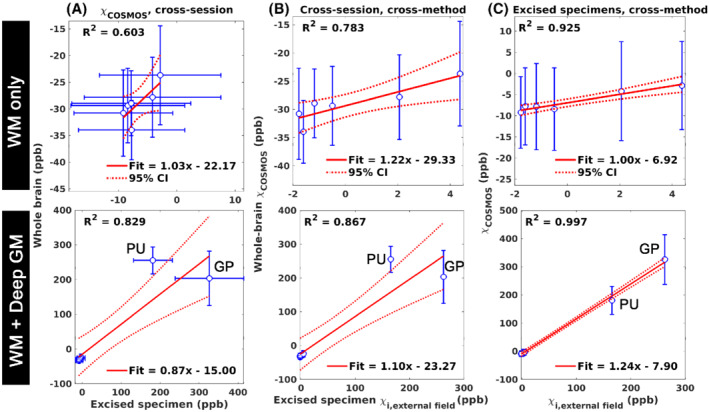
Linear regression analyses with (top) only the six white matter (WM) specimens and (bottom) when the deep gray‐matter (GM) specimens are included. The magnetic susceptibility measured between two imaging sessions using COSMOS (A), between the bulk magnetic susceptibility measured by COSMOS in the first session and the χ_i_ from the external field measurement in the second session (B), and the excised specimens between COSMOS magnetic susceptibility and external field derived χ_i_ (C). Blue dots: measurement data; solid red line, fitted line; dashed line, 95% CI; error bar, SD

Strong linear relationships were found in χ_i_ estimated by COSMOS between the excised specimens and the corresponding regions of interest in the whole‐brain data (cross‐session; R^2^ = 0.603, Figure 4A), between the χ_i_ of external field measurement and the χ_i_ of the whole‐brain COSMOS (cross‐session, cross‐method; R^2^ = 0.783; Figure 4B), and between the χ_i_ of external field measurement and the COSMOS χ_i_ on the excised specimens (cross‐method; R^2^ = 0.925; Figure 4C). All slopes of the linear regressions are close to 1, whereas the large intercepts in Figure 4A,B reflect the different reference media in the scans (first session: water; second session: 1% agar).

## 4 DISCUSSION

In this study, we examined the magnetic susceptibility and the microstructural compartmentalization effect on a formalin‐fixed, postmortem human brain specimen using GRE phase imaging. The bulk magnetic susceptibility of the whole‐brain specimen shows that WM is slightly diamagnetic, whereas cortical and deep GM are paramagnetic, as observed in both postmortem studies^4^, ^25^ and in vivo imaging.

The COSMOS residual fields of the postmortem data show two interesting properties. The residual fields are relatively constant across different rotations. This differs from the in vivo imaging results (Figures S4 and S5), where the magnetic susceptibility differences are more pronounced, suggesting a reduced effect of (sub)cellular structure of WM and that the sphere of Lorentz inclusion used in COSMOS is justified in this fixed sample. Second, the residual fields have a monotonous change (increase) from the brain surface to deeper tissues (Figure 2E), corresponding to the distance that formaldehyde diffuses into the tissues. This effect may reflect the degree of tissue degradation, as the fixative takes longer to diffuse into the deeper tissues with a sufficient quantity, and autolysis may have occurred when deeper tissues were not yet fixed.

Although the χ_i_ of WM samples remained similar to in vivo, the analysis of the residual fields inside the excised tissues confirmed that the microstructure compartmental frequency (parameter A in Eq. 4) in our formalin‐fixed samples is notably weaker than in vivo and reported by others. In a similar experiment,^43^ the amplitude of the microstructure frequency of a fresh bovine optic nerve at 7 T was −18.75 ppb, which is significantly larger in magnitude and with an opposite sign to what we obtained in our samples (1.46 ppb). A reduction in the microstructural compartmentalization effect has already been reported elsewhere.^26^ A reduction of the orientation dependence of R_2_* with respect to in vivo measurements^45^ was also observed (see Supporting Information Section 3.2). One possible explanation is the structural change in the myelin sheath in fixed tissues. We performed an additional analysis using 3D electron microscopy on two excised WM specimens to understand the MRI findings (see Supporting Information Section 4). We observed splitting and swelling of the myelin sheath in some of the myelinated axons, which were also found in a previous report.^46^ Such phenomena were dominant in larger axons. The general Lorentzian tensor approach^47^ predicts that an enlargement of the aqueous space of the myelin sheath leads to a reduction of the induced frequency shift amplitudes within the myelin sheath and the intra‐axonal space. Microstructural differences in terms of structure (bovine optic nerve vs human CC), age‐associated demyelination,^48^ and magnetic field strength (7T vs 3T in this work), together with the tissue preparation methods, could also influence the relative relaxation rates of myelin and intra/extra‐axonal water and contribute to the differences observed in this study.

All specimens from the body of the CC have similar χ_a_, suggesting that they have similar myelin water fraction (MVF) based on the hollow cylinder fibre model (HCM) approximation (Eq. S25 in Wharton and Bowtell^15^), and this is supported by the electron microscopy analysis (0.278 and 0.257 between the two samples; Table S1). The amplitude of the residual field within the sample, on the other hand, is subject to various properties, including MVF, axonal volume fraction, and the aggregated g‐ratio (Eq. A14 in Wharton and Bowell^14^). Interestingly, the realistic geometry of the WM fiber can also play an important role in the compartmental frequency shifts (Figure S8 and Table S1)^12^, ^15^, ^28^, ^49^, ^50^, ^51^: Not only the mean but also the FWHM of the extracellular frequency distribution of the two samples are different, despite the two specimens having nearly identical MVF and axonal volume fraction. The broader frequency spectrum of CC4 induces a faster R_2_* decay in the extra‐axonal space, and the specimen also has a more dispersed fiber arrangement. These two factors can reduce the amplitude discrepancy between the slow R_2_* (intra‐axonal and extra‐axonal water) and fast R_2_* (myelin water) compartments and their frequency difference, which could cause a reduced compartmentalization effect.

This study demonstrates the shortcomings of standard fixation techniques on large brain samples when examining WM microstructure using GRE. Experiments performed with a shorter fixation time could reduce the fixation effects on the signal phase—similar to what is observed with the R_1_ measurements shown in Figure 2 (R_1_ of WM using MP2RAGE were about 1.2 s^−1(^ ^52^ ^)^, 1.95 s^−1^, and 2.7 s^−1^ for in vivo imaging, after 1‐month and after 5‐month fixation periods). However, it is preferable to have a fixation period of at least 2–3 months so that the MR parameters become stable.^53^ Perfusion fixation would be a solution to reduce autolysis in deeper brain regions.^54^ Alternatively, postmortem in situ imaging does not require formalin fixation^55^ but would require a faster acquisition and sample excision protocols to minimize autolysis during the experiment.

The 3D‐printed holder method used here can be useful in other ex vivo studies that involve histology, as it facilitates tissue excision with high precision by providing landmarks in MRI images for sample matching. This is confirmed by the close‐to‐unity linear relationships of the susceptibility measurements between the two sessions (Figure 4A,B).

## 5 CONCLUSIONS

The contributions of MR phase contrast observed in the postmortem formalin‐fixed brain specimen differ substantially from fresh tissue, despite the resulting QSM maps showing similar contrasts and values to those from in vivo imaging. Particularly, the reductions in magnetic susceptibility anisotropy and compartmentalization are observed in the fixed WM tissue. Therefore, WM magnetic susceptibility and microstructural quantification findings in studies with formalin‐fixed tissue should be interpreted with care. Our study suggests that the microstructural effects observed in our samples encode information about WM arrangements such as dispersion and packing, while susceptibility anisotropy encodes myelin volume, as the theory predicted.

## Supporting information


**Figure S1.** Two example slices demonstrate the registration between the excised specimens and the whole‐brain MRI data. (Left) R_1_ maps that were used to plan the excised specimen experiment and (right) the corresponding slices that were extracted from the whole‐brain specimen. The R_1_ contrast between the holder material and grid spacing filled with water provided a coordinate system that was used directly when planning the tissue excision (bottom left: red squares indicate the elements having relatively high DTI flip angle [FA] ≥ 0.45°), as well as to guide the excision instruments. Every five columns/rows on the plate has a landmark (blue arrows) offering supplementary features to aid in the identification of the coordinates. Because the specimen was fitted tightly in the middle of the plate holder, tissue deformation during the tissue extraction process was substantially reduced, but modest degrees of local deformation and rotation were still possible because of the plasticity of the tissue
**Figure S2.** The experiment setup for the excised specimen magnetic susceptibility measurement. (A) Twelve specimens (10 white matter [WM], 2 deep gray matter [GM]) were excised from the whole‐brain sample and embedded in 1% low‐gelling temperature agarose on two levels (six specimens for each level, black arrows). The predefined rotation angles and acquisition sequence were uniformly marked onto the container surface (red arrows), providing coarse signs to guide the rotations. The actual angles of rotation and the angles between the fiber orientation and the B_0_ directions used in the analysis of this work were derived from the transformation matrices of the image registration. (B) Plot showing the main direction of the WM samples with respect to B_0_ (in acquisition 6, samples were aligned along with B_0_). The color of the vector represents the temperature variation across the 10 gradient‐echo (GRE) acquisitions. The temperature was measured before each new rotation via an external container of comparable size and filled with water (positioned next to the container with the specimens but outside the head/neck coil). The actual rotation angles with respect to orientation 6 are (in the acquisition order) [50, 60, 9, 66, 39, 0, 18, 31, 81, 88]°
**Figure S3.** B_0_ directions from the head/specimen rotation on (left) QSM challenge 1 in vivo data set (12 acquisitions) and (right) the formalin‐fixed postmortem specimen for this work (10 acquisitions). The blue markers denote the subset of data for the comparison in section 2.2. The black reference vector (Ref.) on the right represents the common space in which data from all orientations were registered. The rotation angles with respect to the reference for the whole‐brain imaging session of this work are [12, 12, 36, 46, 61, 62, 75, 77, 78]°
**Figure S4.** Calculation of susceptibility through multiple orientation sampling (COSMOS) and quantitative susceptibility and residual (QUASAR) results on (left) in vivo imaging data set from QSM challenge 1 (12 rotations in total) and (right) formalin‐fixed postmortem brain specimen (10 rotations). (From top to bottom) Bulk magnetic susceptibility (χ) maps derived by COSMOS, χ maps derived from QUASAR, differences between the COSMOS and QUASAR χ maps, and nonsusceptibility contribution maps derived from QUASAR. The in vivo imaging and ex vivo imaging results show similar image contrasts, with iron‐rich basal ganglia, red nucleus and substantia nigra being the brightest in the maps (blue arrows) and myelin‐rich white matter being the darkest. Interestingly, the in vivo QUASAR‐derived χ map is more homogenous within white matter in contrast to the COSMOS counterpart (red arrows). The contrasts among WM fiber bundles in the χ map can be originated from magnetic susceptibility anisotropy and microstructural difference and are partially explained in the nonsusceptibility contribution map of QUASAR. On our formalin‐fixed specimen, the difference in χ between COSMOS and QUASAR is considerably smaller than on those taken from the in vivo data set
**Figure S5.** (Top row) COSMOS‐derived magnetic susceptibility maps on the in vivo and postmortem data using a subset of four orientations (see Figure S3). Despite being noisier, the magnetic susceptibility maps from the two data sets still share similar image contrasts with each other, and with the full data set. (Bottom 4 rows) Residual field of each orientation. It is clear that there is a persistent positive residual toward the deeper tissue on the formalin‐fixed specimen, which is absent in the in vivo results
**Figure S6.** Results of fitting the isotropic and anisotropic magnetic susceptibility of the excised white matter (WM) tissue specimens with and without a constant term to account for acquisition difference (eg, shimming) for each orientation. Blue lines with triangular markers represent the mean residual fields of the susceptibility computation in the external agar region without considering the constant term; light blue lines with cross markers represent the mean residual fields in the same region with the constant term being considered; orange lines represent the fitted constant terms. Note that the orange lines have an identical shape as the mean residual field when the constant term was not included in the fitting, and the mean residual fields are close to zero once we introduced this term in the fitting
**Figure S7.** Data fitting of the R_2_*(*θ*) = Asin^2^ *θ*+B function, based on the average R_2_* of the specimens, and *θ* is the angle between the main sample fiber orientation and the B_0_ direction, similar to Figure 3. The mean value of A, representing the maximum orientation dependence of R_2_* (ie, $\Delta R_{2}^{*}=R_{2,⊥}^{*}-R_{2,∥}^{*}$) across CC1–CC6 is 1.31 Hz (ranging from 0.31 to 1.96 Hz), which is weaker than those observed in vivo at 3 T (3–5 Hz; see Figures 5 and 6 of Rochefort et al^1^)
**Figure S8.** Frequency induced by the myelin χ_i_ at two orientations to B_0_ (*θ* = 0° and 90°). Subfigures show the histograms of the frequency distributions in extracellular space (x‐axis: frequency range of the distribution; y‐axis: probability). The locations and the FWHM of the peaks are indicated in Table S1
**Table S1.** (Top) Summary of the 3D extracellular matrix (EM)–derived CC4 and CC5 microstructural properties. Note: The myelin water fraction (MVF) was probed separately using five different intensity thresholds (only the middle value indicated by * was shown in the g‐ratio). The corrected MVF was derived using Eq. S1.
