# Supplementary material for: Imaging white matter microstructure with gradient‐echo phase imaging: Is ex vivo imaging with formalin‐fixed tissue a good approximation of the in vivo brain?
Source: Magn Reson Med. 2022 Mar 28;88(1):380–90. doi: 10.1002/mrm.29213 (PMC9314807; doi:10.1002/mrm.29213)
Supplement: Supplementary file 1 — Figure S1. Two example slices demonstrate the registration between the excised specimens and the whole‐brain MRI data. (Left) R1 maps that were used to plan the excised specimen experiment and (right) the corresponding slices that were extracted from the whole‐brain specimen. The R1 contrast between the holder material and grid spacing filled with water provided a coordinate system that was used directly when planning the tissue excision (bottom left: red squares indicate the elements having relatively high DTI flip angle [FA] ≥ 0.45°), as well as to guide the excision instruments. Every five columns/rows on the plate has a landmark (blue arrows) offering supplementary features to aid in the identification of the coordinates. Because the specimen was fitted tightly in the middle of the plate holder, tissue deformation during the tissue extraction process was substantially reduced, but modest degrees of local deformation and rotation were still possible because of the plasticity of the tissue Figure S2. The experiment setup for the excised specimen magnetic susceptibility measurement. (A) Twelve specimens (10 white matter [WM], 2 deep gray matter [GM]) were excised from the whole‐brain sample and embedded in 1% low‐gelling temperature agarose on two levels (six specimens for each level, black arrows). The predefined rotation angles and acquisition sequence were uniformly marked onto the container surface (red arrows), providing coarse signs to guide the rotations. The actual angles of rotation and the angles between the fiber orientation and the B0 directions used in the analysis of this work were derived from the transformation matrices of the image registration. (B) Plot showing the main direction of the WM samples with respect to B0 (in acquisition 6, samples were aligned along with B0). The color of the vector represents the temperature variation across the 10 gradient‐echo (GRE) acquisitions. The temperature was measured before each new rotation via an external [file MRM-88-380-s001.docx]

# Supporting Information

## Section 1: Additional information on the experiment procedure

### 1.1. Registration of the excised specimens to their corresponding locations in the whole-brain data

Figure S1: Two example slices to demonstrate the registration between the excised specimens and the whole-brain MRI data. (Left) R_1_ maps that were used to plan the excised specimen experiment and (right) the corresponding slices that were extracted from the whole-brain specimen. The R_1_ contrast between the holder material and grid spacing filled with water provided a coordinate system that was used directly when planning the tissue excision (bottom left, red squares indicated the elements having relatively high DTI’s FA≥0.45), as well as to guide the excision instruments. Every 5 columns/rows on the plate has a landmark (blue arrows) offering supplementary features to aid in the identification of the coordinates. Since the specimen was fitted tightly in the middle of the plate holder, tissue deformation during the tissue extraction process was substantially reduced, but modest degrees of local deformation and rotation were still possible because of the plasticity of the tissue.


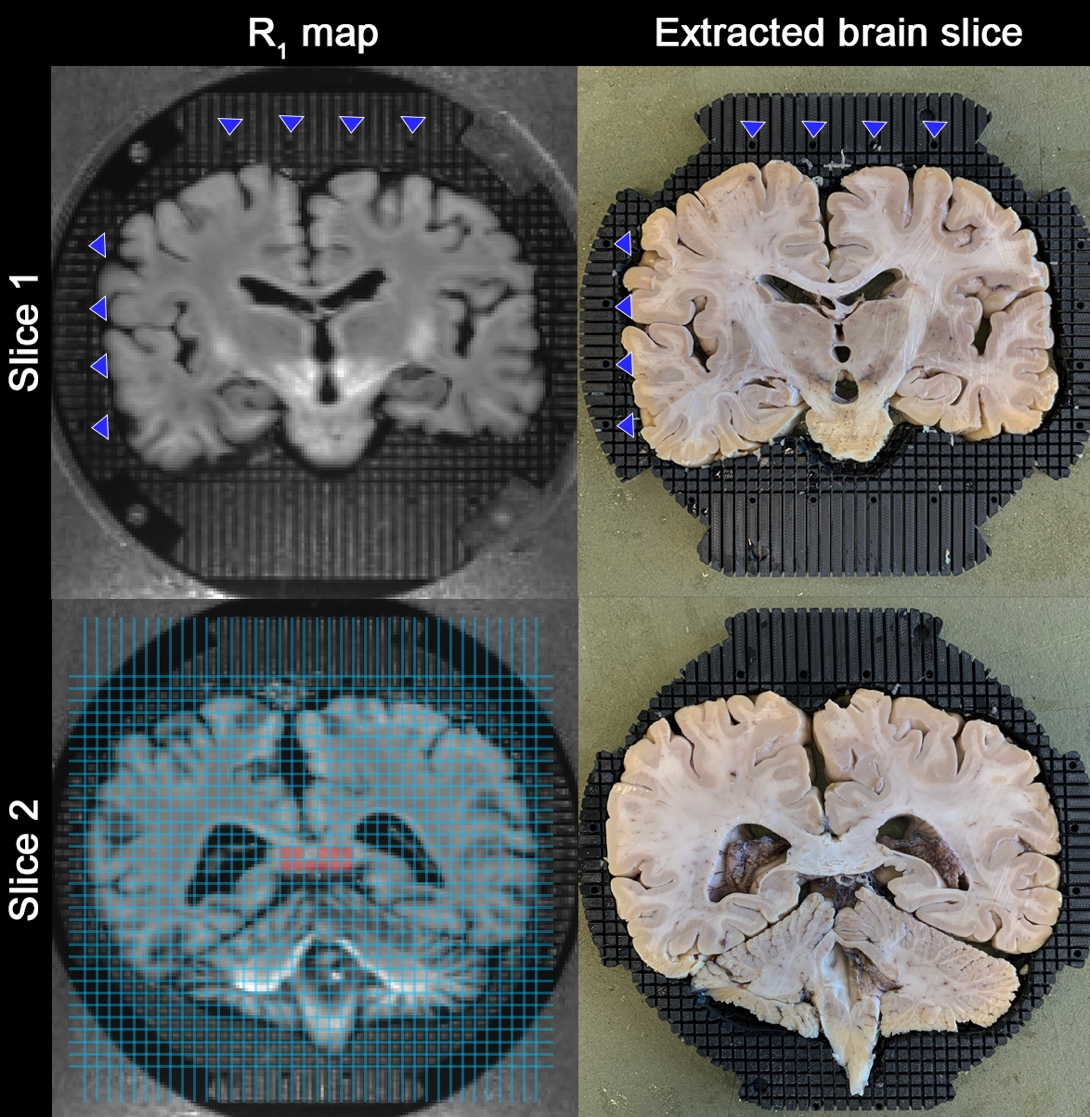


### 1.2. The excised specimen experiment set-up

Figure S2: The experiment set-up for the excised specimen magnetic susceptibility measurement. (A) Twelve specimens (10 WM, 2 deep GM) were excised from the whole-brain sample and embedded in 1% low-gelling temperature agarose on two levels (6 specimens for each level, black arrows). The pre-defined rotation angles and acquisition sequence were uniformly marked onto the container surface (red arrows) providing coarse signs to guide the rotations. The actual angles of rotation and the angles between the fibre orientation and the B_0_ directions used in the analysis of this work were derived from the transformation matrices of the image registration. (B) Plot showing the main direction of the white matter samples with respect to B_0_ (in acquisition #6 – samples were aligned along with B_0_). The colour of the vector represents the temperature variation across the 10 GRE acquisitions. The temperature was measured before each new rotation via an external container of comparable size and filled with water (positioned next to the container with the specimens but outside the head/neck coil). The actual rotation angles with respect to orientation #6 are (in the acquisition order): [50, 60, 9, 66, 39, 0, 18, 31, 81, 88]°.


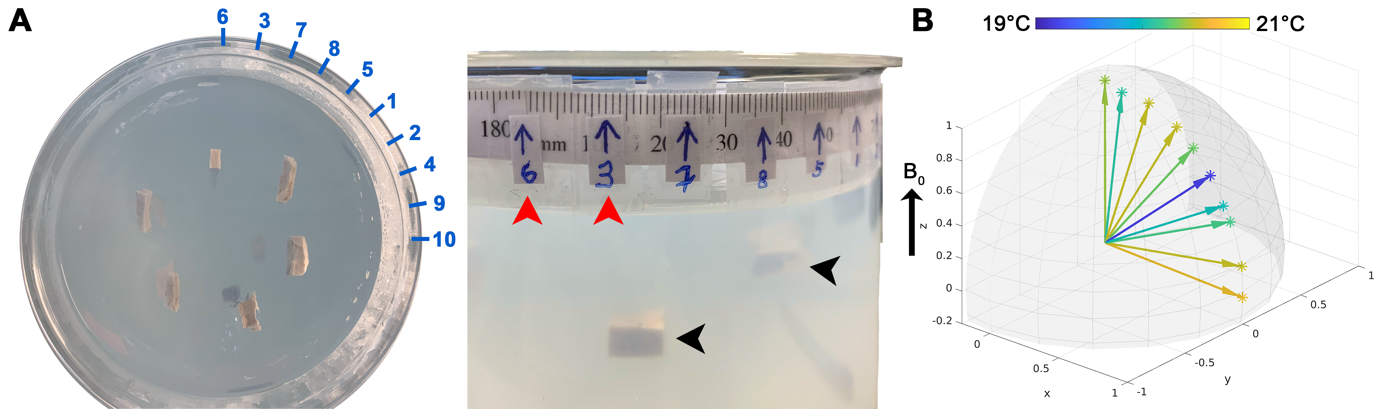


## Section 2: Whole-brain imaging comparison to in vivo imaging dataset

Figure S3: B_0_ directions from the head/specimen rotation on (left) QSM challenge 1 in vivo dataset (12 acquisitions) and (right) the formalin-fixed post-mortem specimen for this work (10 acquisitions). The blue markers denote the subset of data for the comparison in sub-section 2.2. The black reference vector (Ref.) on the right represents the common space in which data from all orientations were registered. The rotation angles with respect to the reference for the whole-brain imaging session of this work are: [12, 12, 36, 46, 61, 62, 75, 77, 78]°.


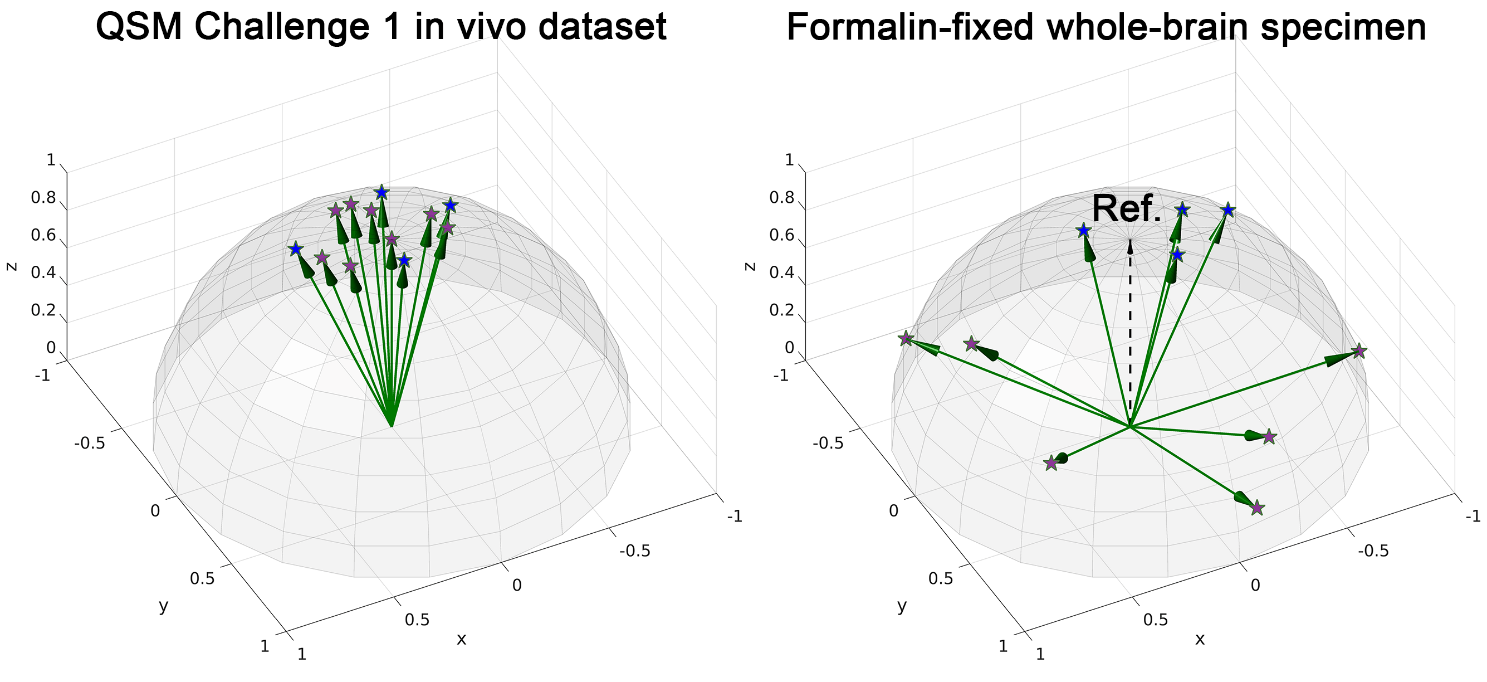


### 2.1. Applying COSMOS and QUASAR on the full set of acquisition

Figure S4: COSMOS and QUASAR results on (left) in vivo imaging dataset from QSM challenge 1 (12 rotations in total) and (right) formalin-fixed post-mortem brain specimen (10 rotations). (From top to bottom) Bulk magnetic susceptibility (𝜒) maps derived by COSMOS, 𝜒 maps derived from QUASAR, differences between the COSMOS and QUASAR 𝜒 maps, and non-susceptibility contribution maps derived from QUASAR. The in vivo imaging and ex vivo imaging results show similar image contrasts, with iron-rich basal ganglia, red nucleus and substantia nigra being the brightest in the maps (blue arrows) and myelin-rich white matter being the darkest. Interestingly, the in vivo QUASAR derived 𝜒 map is more homogenous within white matter in contrast to the COSMOS counterpart (red arrows). The contrasts among WM fibre bundles in the 𝜒 map can be originated from magnetic susceptibility anisotropy and microstructural difference and are partially explained in the non-susceptibility contribution map of QUASAR. On our formalin-fixed specimen, the difference in 𝜒 between COSMOS and QUASAR is considerably smaller than on those taken from the in vivo dataset.


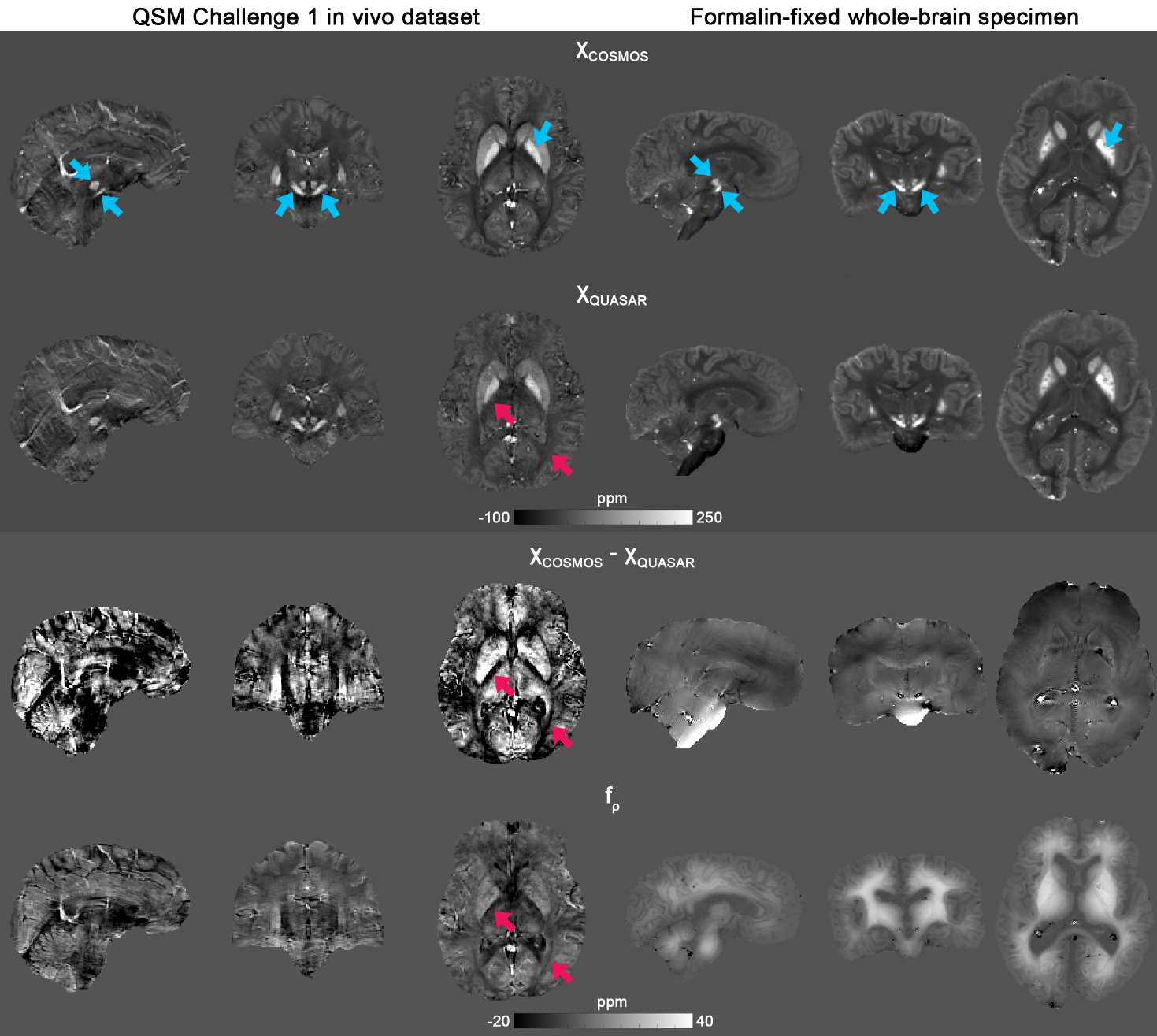


### 2.2. Applying COSMOS with a subset of acquisition that have similar relative orientations

Figure S5: (Top row) COSMOS-derived magnetic susceptibility maps on the in vivo and post-mortem data using a subset of 4 orientations (see Figure S3). Despite being noisier, the magnetic susceptibility maps from the two datasets still share similar image contrasts with each other, and with the full dataset. (Bottom 4 rows) The residual field of each orientation. It is clear that there is a persistent positive residual toward the deeper tissue on the formalin-fixed specimen which is absent in the in vivo results.


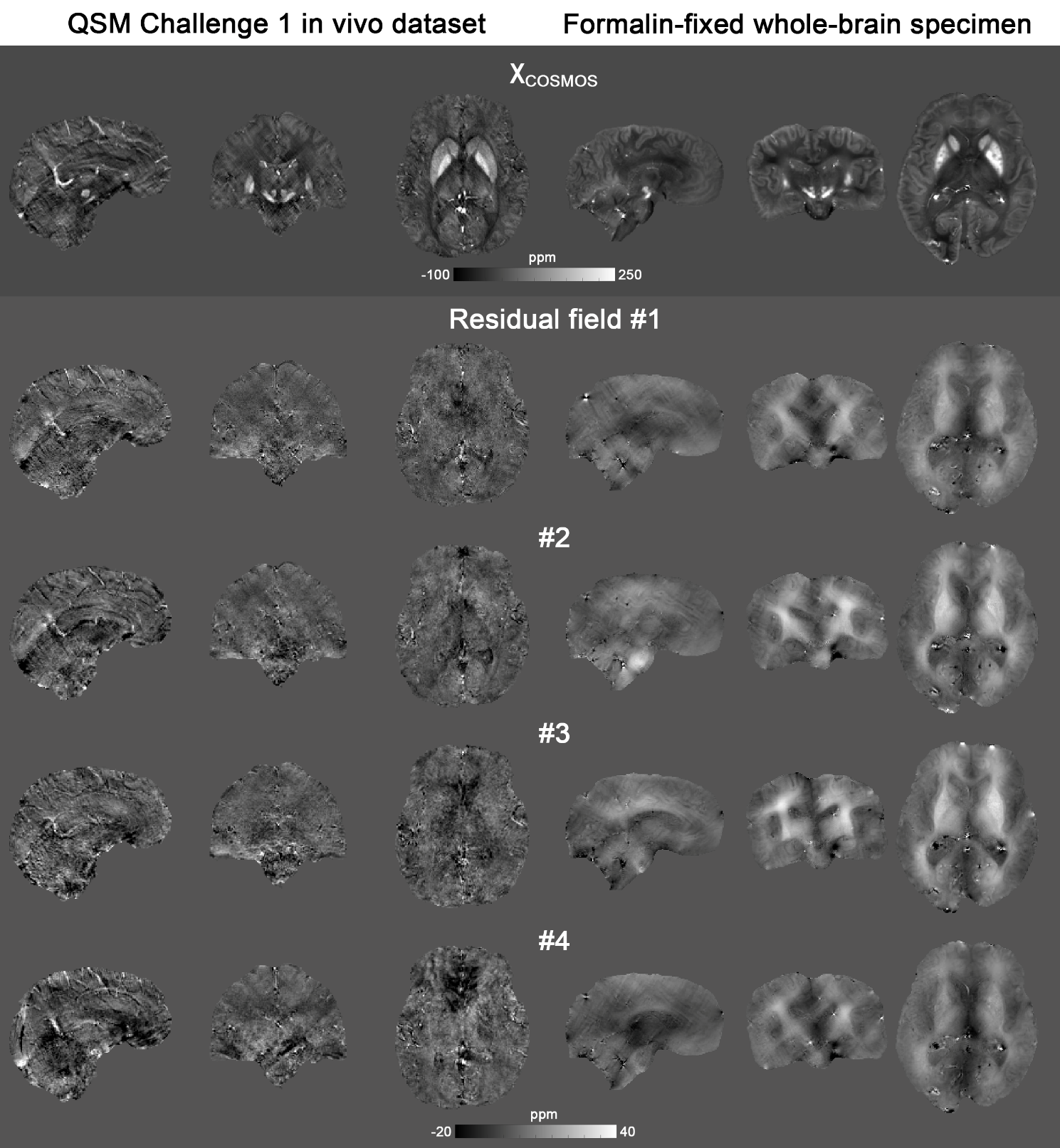


## Section 3: Supplementary analyses on the excised specimens

### 3.1 Measurement of isotropic and anisotropic magnetic susceptibility based on the external field of the white matter specimens

Figure S6: Results of fitting the isotropic and anisotropic magnetic susceptibility of the excised WM tissue specimens with and without a constant term to account for acquisition difference (e.g., shimming) for each orientation. **Blue lines with triangular markers** represent the mean residual fields of the susceptibility computation in the external agar region **without considering the constant term**; **light blue lines with cross markers** represent the mean residual fields in the same region **with the constant term being considered**; orange lines represent the fitted constant terms. Note that the orange lines have an identical shape as the mean residual field when the constant term was not included in the fitting, and the mean residual fields are close to zero once we introduced this term in the fitting.


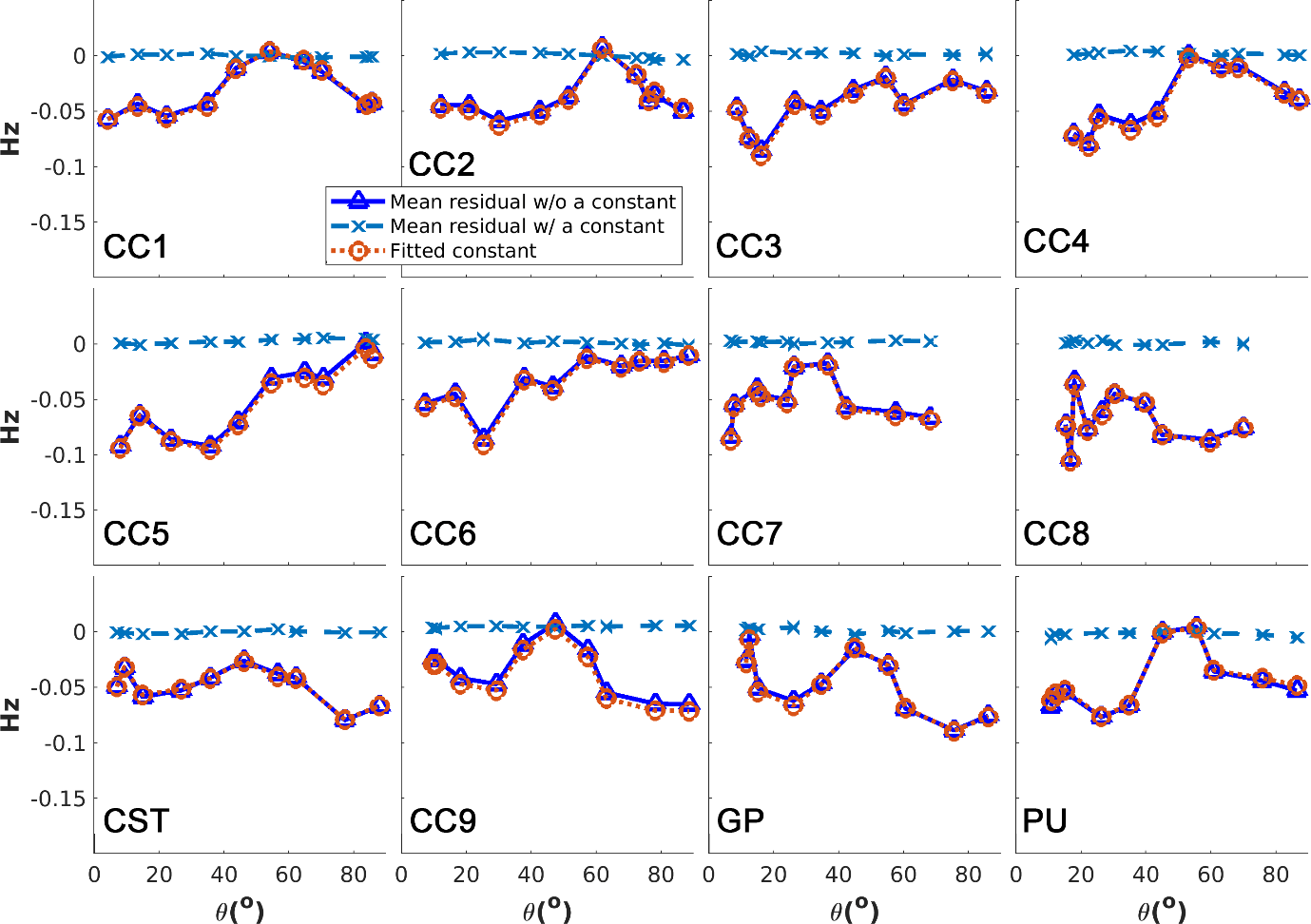


### 3.2 Measurement of the R_2_* orientation dependence on the excised specimens

Figure S7: Data fitting of the R_2_*(𝜃)=Asin^2^𝜃+B function, based on the average R_2_* of the specimens and 𝜃 is the angle between the main sample fibre orientation and the B_0_ direction, similar to Figure 3. The mean value of A, representing the maximum orientation dependence of R_2_* (i.e., $\Delta R_{2}^{*}=R_{2,\perp}^{*}-R_{2,\parallel}^{*}$), across CC1-CC6 is 1.31Hz (ranging from 0.31 to 1.96Hz), which is weaker than those observed in vivo at 3T (3-5Hz see Figures 5 and 6 of Reference (1)).


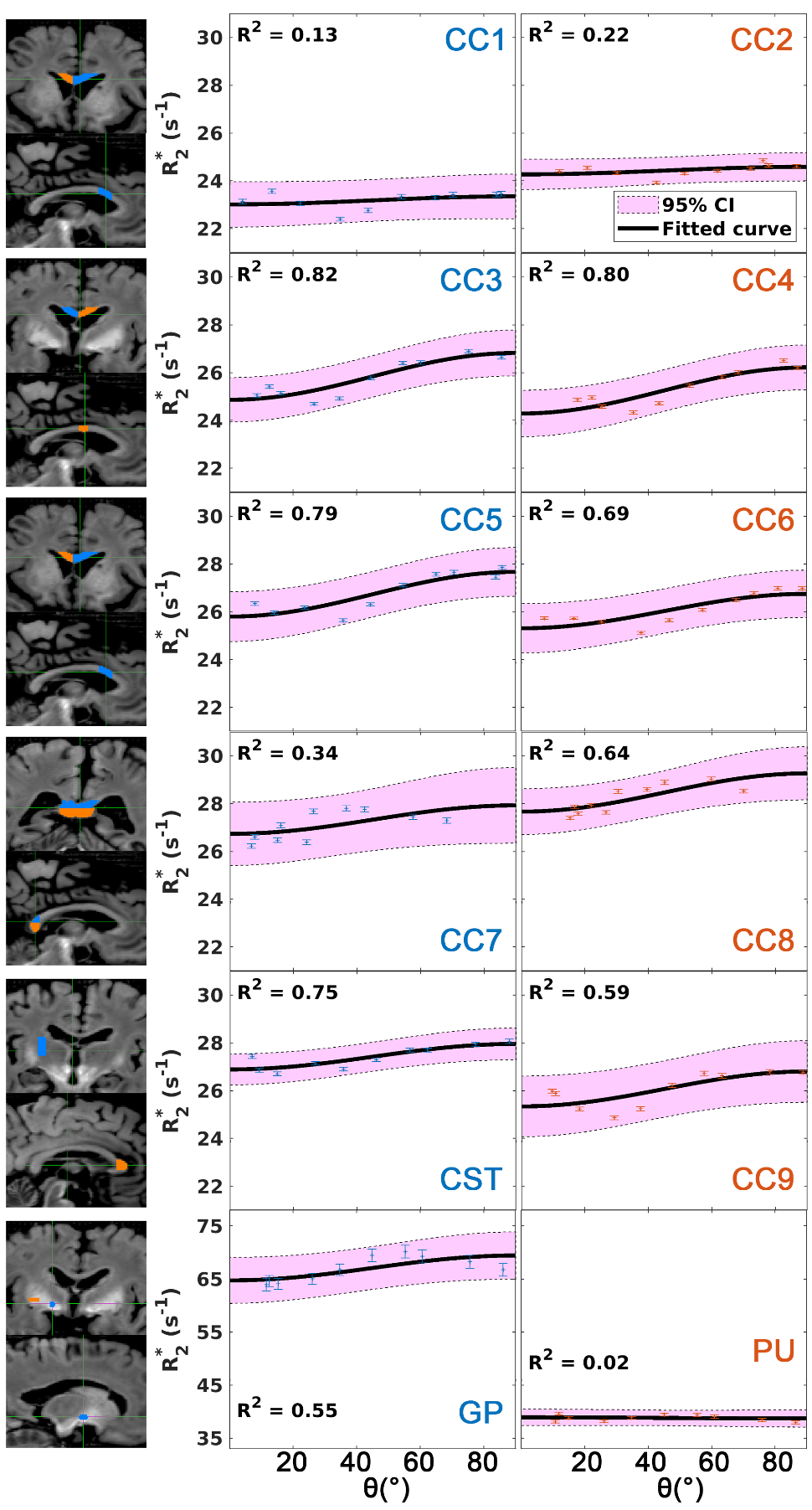


## Section 4: 3D Electron microscopy for microstructure analysis

We utilised 3D electron microscopy to provide an additional reference to understand and explain the MRI findings.

### **Tissue preparation**

Two days after the second MRI session, the WM specimens were sectioned to 100 𝜇m on a vibratome (VT1000S, Leica Biosystems, Nussloch, Germany) before being immersed in 2.5% glutaraldehyde in 0.1M sodium cacodylate buffer for overnight incubation at 4°C. The specimens were then transferred to 0.25% glutaraldehyde in 0.1M sodium cacodylate buffer for storage at 4°C and then delivered to the EM facility at the University of Oxford for imaging.

### **Data acquisition**

Two corpus callosum (CC) specimens from the second MRI session (CC4 and CC5) having the greatest discrepancy of the microstructural compartmentalisation effect underwent 3D EM (2) to provide histology data for the MRI experiment validation. Each of the EM images has a matrix size of 8000×8000 with an in-plane resolution of 13.7 nm ×13.7 nm (~0.11 mm x 0.11 mm FOV) and slice thickness of 100 nm. In total, 651 and 623 slices were acquired for CC4 and CC5 respectively.

### **Data processing**

Three-compartment microstructure classification

Our EM data showed similar myelin sheath damage (splitting and swelling) as illustrated in (3), resulting in unsatisfactory compartmental classification (intra-axonal, myelin and extra-axonal compartments) using standard segmentation tools. To obtain high-quality 3-compartment classification, we first performed a semi-automatic intra-axonal segmentation using ITK-snap (4) on down-sampled 3D EM data (in-plane resolution=87.7𝜇m×87.7𝜇m, matrix size=1250×1250, and only 100 consecutive slices were used). The myelin sheath of each axon was initially defined by expanding the axon mask, assuming a g-ratio = 0.5 (for axon diameters<1.2𝜇m) or 0.6 (otherwise), followed by intensity thresholding on the EM images (a.u.). The resulting myelin mask was clearly influenced by the chosen threshold, therefore, myelin volume fractions (MVF) derived from 5 threshold values between 125 to 145. The most frequent intensities in the myelin mask were 104 (CC4) and 88 (CC5)). To account for the enlarged myelin volume due to swelling, we further convert the myelin mask into a myelin probability map using the EM image intensity as weights:

:

$$Myelin probability(r)=\left\{ \begin{aligned} 1, &I(r)<M \\ \frac{A-I\left( r \right)}{A-M}, &M<I(r)\leq A \\ 0, &I(r)>A \end{aligned} \right. [Eq. S1]$$

where A and M are the most frequent values inside the axonal and myelin masks and I(r) is the EM image intensity of a voxel. The axon mask and myelin mask of each myelinated axon were combined in a single classification map to avoid repeated counts due to overlapping between neighbouring axons, and the unsegmented area was defined as the extra-axonal space.

Summary statistics of compartmental classification

Axonal volume fraction (AVF) and MVF were computed by counting the total number of voxels of each compartment in the classification map (only 1000×1000x40 voxels in the middle of the 3D EM volume were included to avoid incomplete segmentation close to the edges), and were used to derive the sample g-ratio (5). Effective axonal diameter was defined as the square root of the product of the 2^nd^ and 3^rd^ principal axis lengths of the axons obtained from the *regionprop3* function of MATLAB (Mathworks, Natick, US), from which the median and the skewness of the axonal diameter distribution were computed. Fibre dispersion was computed from the axonal volume-weighted average squared dot product between the axon main principal direction and the average orientation of the entire sample (6).

Field perturbations from isotropic magnetic susceptibility of myelin sheath

To further investigate the effect of realistic myelin sheath geometry on the compartmental frequency shifts, the aforementioned myelin mask was used to simulate field perturbations induced by isotropic magnetic susceptibility (𝝌_i_) of myelin in two scenarios: when the segmented sample axons were parallel or perpendicular to B_0_. This was done by convoluting the myelin mask with a dipole field, and 𝝌_i_ was set to -0.1ppm (7). The fibre direction of each specimen was computed via the log-Euclidean averaging of the 1^st^ principal axis of the axons (obtained from *regionprop3*) and then was used to derive the B_0_ directions parallel and perpendicular to the fibre. This method was previously used in (6), but here we considered only the effect of the magnetic isotropic field for simplicity. The frequency shift distribution in the extracellular space was subsequently analysed in the middle 1000×1000x40 voxels of the 3D EM volume (neglecting the anisotropic component has a smaller effect).

### **Results**

The microstructural properties of two WM specimens (CC4: relatively weaker microstructural phase; CC5: strong microstructural phase) derived from 3D EM data are summarised in Table S1. Both specimens have similar MVF (8% difference), AVF (4% difference) and axonal diameter (1% difference). Noticeable differences are observed in fibre dispersion (50% difference) which result in a large FWHM of the extra-axonal frequency distributions when the fibre direction is parallel to B_0_ (63% difference, Figure S8).


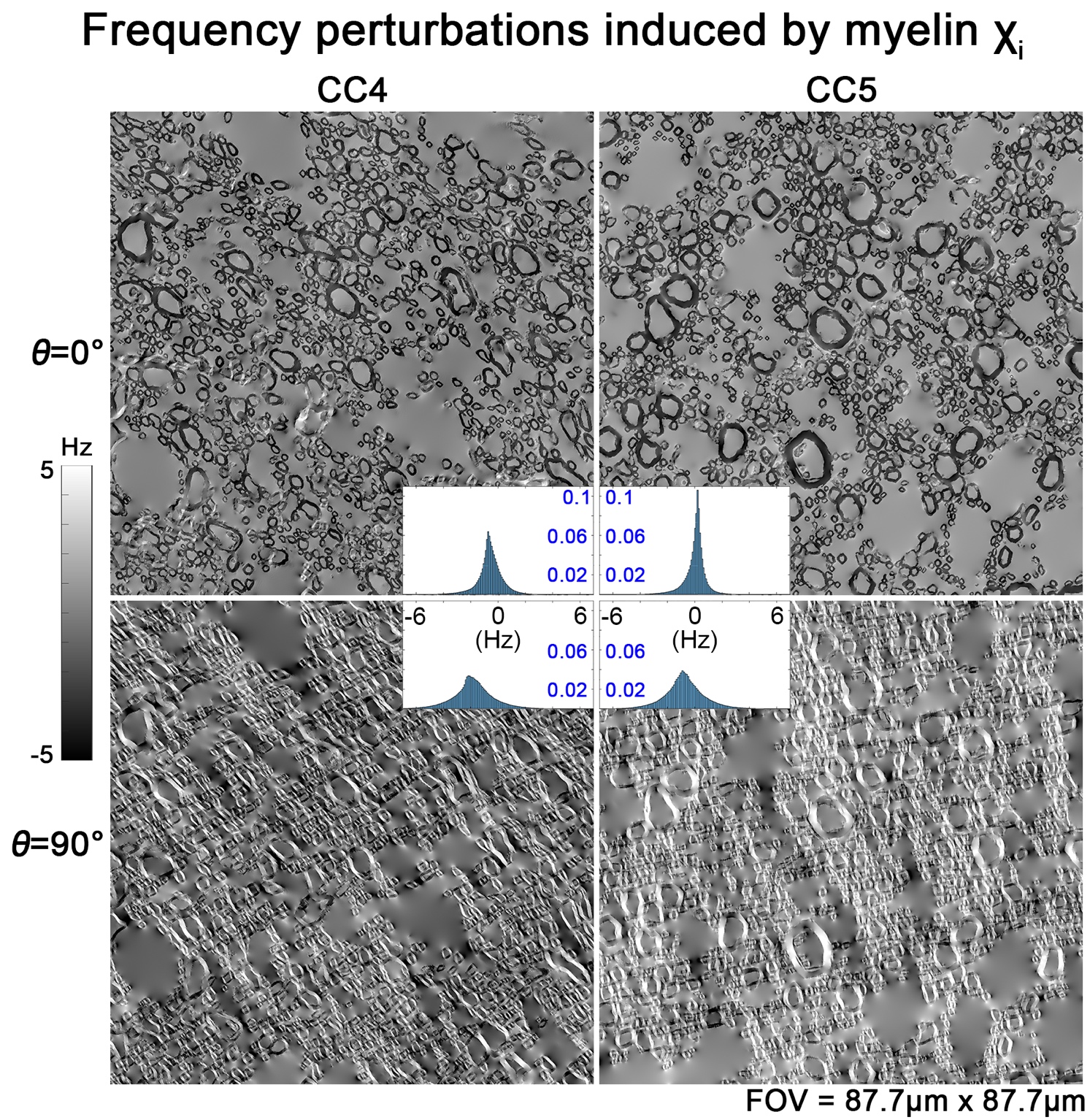


Figure S8: Frequency induced by the myelin 𝜒_i_ at two orientations to B_0_ (𝜃=0° & 90°). Sub-figures show the histograms of the frequency distributions in extracellular space (x-axis: frequency range of the distribution; y-axis: probability). The locations and the FWHM of the peaks are shown in Table S1.

Table S1: (Top) Summary of the 3D EM derived CC4 and CC5 microstructural properties. MVF was separately probed using 5 different intensity thresholds (only the middle value indicated by * was showed in g-ratio). The corrected MVF was derived using Eq. [S1].

|  |  | **CC4** | **CC5** |
| --- | --- | --- | --- |
| **# of Axons** | | 1607 | 1565 |
| **Effective axonal diameter** | **Median, 𝜇m** | 0.944 | 0.957 |
|  | **Skewness** | 1.798 | 2.058 |
| **Dispersion** | | 0.135 | 0.081 |
| **AVF** | | 0.183 | 0.192 |
| **MVF** | | 0.272, 0.304, 0.328*, 0.345, 0.357 | 0.283, 0.304, 0.321*, 0.334, 0.344 |
| **MVF corrected** | | 0.245, 0.265, 0.278*, 0.285, 0.289 | 0.238, 0.249, 0.257*, 0.261, 0.264 |
| **g-ratio** | | 0.599 | 0.611 |
| **g-ratio corrected** | | 0.630 | 0.654 |
| **Peak of extracellular frequency shift caused by myelin 𝝌_i_ (FWHM), Hz** | **𝜽=0°** | -0.735 (0.829) | 0.194 (0.434) |
|  | **𝜽=90°** | -2.189 (1.85) | -0.919 (1.834) |

# References

1. Gil R, Khabipova D, Zwiers M, Hilbert T, Kober T, Marques JP. An in vivo study of the orientation-dependent and independent components of transverse relaxation rates in white matter. NMR in biomedicine 2016;29:1780–1790 doi: 10.1002/nbm.3616.

2. Kleinnijenhuis M, Johnson E, Mollink J, Jbabdi S, Miller KL. A semi-automated approach to dense segmentation of 3D white matter electron microscopy. Biorxiv 2020:2020.03.19.979393 doi: 10.1101/2020.03.19.979393.

3. Duijn S van, Nabuurs RJA, Rooden S van, et al. MRI artifacts in human brain tissue after prolonged formalin storage. Magnetic resonance in medicine 2011;65:1750–1758 doi: 10.1002/mrm.22758.

4. Yushkevich PA, Piven J, Hazlett HC, et al. User-guided 3D active contour segmentation of anatomical structures: Significantly improved efficiency and reliability. Neuroimage 2006;31:1116–1128 doi: 10.1016/j.neuroimage.2006.01.015.

5. Stikov N, Campbell JSW, Stroh T, et al. In vivo histology of the myelin g-ratio with magnetic resonance imaging. NeuroImage 2015;118:397–405 doi: 10.1016/j.neuroimage.2015.05.023.

6. Hédouin R, Metere R, Chan K-S, et al. Decoding the microstructural properties of white matter using realistic models. Neuroimage 2021;237:118138 doi: 10.1016/j.neuroimage.2021.118138.

7. Wharton S, Bowtell R. Fiber orientation-dependent white matter contrast in gradient echo MRI. Proceedings of the National Academy of Sciences 2012;109:18559–18564 doi: 10.1073/pnas.1211075109.
